# Editorial: The role of the pancreas in poultry

**DOI:** 10.3389/fphys.2024.1463203

**Published:** 2024-08-28

**Authors:** Vladimir Vertiprakhov, Alena Grozina, Vladimir Fisinin

**Affiliations:** ^1^ Moscow Timiryazev Agricultural Academy, Moscow, Russia; ^2^ All-Russian Scientific Research and Technological Institute of Poultry, Russian Academy of Agricultural Sciences, Moscow, Russia

**Keywords:** trypsin, pancreas, poultry, taste sensations, feed nutrition

The pancreas in poultry secretes continuously a secretion that hydrolyzes proteins, fats and carbohydrates to monomers that can be absorbed into the blood (Batoev, 2001; Batoev, 2018; Berdnikov et al., 2009). Both during fasting and after a meal, pancreatic secretion is regulated by nervous and hormonal pathways (Pavlov, 1951). Feeding serves as a powerful stimulator of pancreatic secretion. The period of reflex phase regulation of pancreatic secretion in broiler chickens begins in the first minutes of feed intake and lasts up to 90 min from the moment of feeding (Vertiprakhov et al., 2018). At this time, the regulation of the pancreas is provided reflexively, and the feed consumed by the bird ends up in the goiter or stomach, and pancreatic juice is intensively secreted into the intestine under the influence of impulses arriving via parasympathetic fibers from the digestive center in the medulla oblongata, where they come from the receptors of the oral cavity (Vertiprakhov and Egorov, 2016). In this case, depending on the strength of the stimulus acting on oral receptors, the level of pancreatic secretion changes, i.e., this period is conditioned by gustatory sensations from the quality of feed. Feed intake increases pancreatic juice secretion 2–3 times compared to the preprandial period (Vertiprakhov and Ovchinnikova; Vertiprakhov et al.).

Studies on chickens and other birds have shown that the avian gustatory system consists of taste buds that are not assembled in papillae but are located mostly (60%) in the upper palate, hidden in the crevices of salivary ducts (Liu et al., 2018). Chickens appear to have an acute sense of taste that allows them to distinguish food amino acids, fatty acids, sugars, quinine, calcium and salt among others. A chemosensory system linked to the enteroendocrine system has been found in the gastrointestinal tract and hypothalamus of birds that mediates a dialog between the gut and the brain relevant to the control of food intake (Cheled-Shoval et al., 2015; Niknafs and Roura, 2018). Experimental data using real-time PCR showed the expression of taste receptor genes in chickens. The expression of these genes suggests the involvement of taste pathways for the perception of carbohydrates, amino acids and bitter compounds in the gastrointestinal tract of chickens. Umami taste is one of the five basic taste qualities, along with sweet, bitter, sour, and salty, and is determined by several amino acids and their salts, including monocalic glutamate (MPG) (Yoshida et al., 2015). The fact that the secretory function of the pancreas in chickens responds to flavor additives to feed is proved by experiments on birds with pancreatic duct fistula (Vertiprakhov, 2022). Thus, the presence of receptors in the bird’s mouth cavity is the beginning of the reflex arc, which is directed to the medulla oblongata and determines the reflex phase of digestion regulation, which lasts from 0 to 60–90 min after feeding in chickens (Vertiprakhov, 2022).

The neurohumoral phase of regulation of pancreatic secretion begins 60–90 min after feeding (Vertiprakhov and Borisenko, 2019), which is associated with the release of secretin and cholecystokinin in the intestine when acidic food enters the intestine from the stomach, which stimulate the release of pancreatic juice and its enzymes (DeGolier et al., 1999). In ducks, a peptide that activates pituitary receptors has been found to be involved in regulation (Wang and Cui, 2007). In the brainstem, vagovagal enteropancreatic reflexes can be modulated by incoming signals from higher brain centers, in particular, the hypothalamic-cholinergic system, with tonic stimulation of preganglionary neurons of the dorsal motor nucleus of the vagus nerve, which affects the function of the pancreas (Singer and Niebergall-Roth, 2009).

The role of pancreatic enzymes in animal blood in the regulation of pancreatic secretion is still a matter of debate. The hypothesis of Laporte and Tremolieres (Laporte and Tremolieres, 1971) is interesting in this respect. Trypsin and chymotrypsin in the duodenum were found to have an inhibitory effect on pancreatic enzyme secretion in rats (Kuzmina et al.). In addition, the feedback mechanism is mediated by neurons including the cholinergic pathway (Lui et al., 1986). Experiments conducted on chickens showed that 60 min after feeding, serum trypsin activity increased, while amylase and lipase activities remained at preprandial levels (Vertiprakhov and Grozina, 2018). A correlation was found in the postprandial period: trypsin levels in the postprandial period increased simultaneously in intestine and blood, in parallel with nitric oxide levels, indicating the participation of pancreatic enzymes in the regulation of pancreatic secretory function in poultry (Fisinin et al., 2018; Vertiprakhov and Ovchinnikova). The increase in trypsin activity closely correlates with the content of deposited NO in blood, which indicates the participation of trypsin in the processes of metabolic regulation in animals and humans along with the parasympathetic nervous system.

It is known that pancreatic juice entering the duodenum contains two pools of enzymes: newly synthesized and reconstituted (Vertiprakhov et al., 2016). The rate of enzyme synthesis does not keep up with exosecretion, which was shown when considering the enzyme-secretory activity of the pancreas (Rothman et al., 2002). Consequently, deficits in enzyme synthesis are compensated for by enzyme reduction. The data on the presence of active digestive enzymes in poultry blood were confirmed by biochemical studies.

The results of the experiment on poultry differ significantly from the data obtained on rats (Laporte and Tremolieres, 1971; Korotko and Baibekova, 1989), which showed an inverse relationship between the content of trypsin in blood and pancreatic juice. Specific bipolar islet-acinar cells with secretory and endocrine function, detected by ultrastructural analysis of the secretory cycle (Permyakov et al., 1973), suggest the possibility of simultaneous entry of trypsinogen into both the pancreatic duct and the bloodstream.

The pancreas performs endocrine function with the involvement of hormones such as insulin and glucagon. Insulin is synthesized in β-cells of the pancreas (Moore and Cooper, 1991; Norton et al., 2022). Insulin secretion is related to metabolic rate in broilers (Vertiprakhov et al., 2023). Tissue metabolism has shown a unique effect of insulin on amino acid metabolism (Qaid et al., 2016).

Insulin is a hormone produced by the pancreas in response to high blood glucose levels. It plays a crucial role in the regulation of glucose metabolism in the body (Saltiel and Kahn, 2001). In chickens, insulin has been shown to have several effects, including the following.

1) Glucose uptake: insulin stimulates the transport of glucose from the bloodstream into cells. In chickens, this is especially important for muscle cells, which use a lot of glucose for energy production during movement and growth (Qaid et al., 2016).
2) Lipid metabolism: insulin also regulates lipid metabolism in chickens by promoting the accumulation of excess fat in adipose tissue and inhibiting the breakdown of stored fat for energy (Saltiel and Kahn, 2001).
3) Growth and development: insulin is a key regulator of chick growth and development, especially in the early stages of life. It promotes cell division and differentiation, resulting in increased muscle and bone growth (Hill and Milner, 1985).
4) Reproduction: insulin also plays a role in reproduction in birds by regulating steroid hormone production and supporting oocyte development (Sliwowska et al., 2014).

## Conclusion

The pancreas is an unpaired parenchymatous organ belonging to glands of mixed secretion, simultaneously performing exocrine and endocrine functions, as well as participating in the regulation of carbohydrate, protein and fat metabolism in tissues. The exocrine (secretory) part of the pancreas produces digestive juice with enzymes (trypsin, lipase, amylase, proteases, etc.), which enter the duodenal lumen through its exit ducts, subsequently entering the blood, where they perform the role of hormone-like substances in the regulation of metabolism, which is confirmed by the expression of trypsin in many organs and tissues, as well as the presence of special PAR-receptors.

## References

[B1] Batoev T. Z. (2001). The digestive physiology of birds. Ulan-ude. Russia: Buryat State University Publ.

[B2] Batoev T. Z. (2018). Adaptation of pancreatic enzymes during the digestion of food of animal and plant origin *Buryat State University bulletin* . Biol. Geogr. 2, 70–75. 10.18101/2587-7143-2018-2-70-75

[B3] Berdnikov P. P. Ryabukha V. A. Dikikh I. P. Fedorova A. O. Smirnova O. V. (2009). Physiological basis for a combined use of zeolite and sodium hypochlorite during growing of chickens. Agric. Biol. 4, 71–74.

[B4] Cheled-Shoval S. L. Druyan S. Uni Z. (2015). Bitter, sweet and umami taste receptors and downstream signaling effectors: expression in embryonic and growing chicken gastrointestinal tract. Poult. Sci. 94 (8), 1928–1941. 10.3382/ps/pev152 26049797

[B5] DeGolier T. F. Place A. R. Duke G. E. Carraway R. E. (1999). Neurotensin modulates the composition of pancreatic exocrine secretions in chickens. J. Exp. Zool. 283 (4-5), 455–462. 10.1002/(sici)1097-010x(19990301/01)283:4/5<455::aid-jez15>3.3.co;2-n-n 10069040

[B6] Fisinin V. I. Vertiprakhov V. G. Titov V.Yu. Grozina A. A. (2018). Dynamics of the activity of digestive enzymes and the content of deposited nitric oxide in the blood plasma of cockerels after feeding. Russ. Physiol. J. named after I.M. Sechenov. 104 (8), 976–983.

[B7] Hill D. J. Milner R. D. (1985). Insulin as a growth factor. Pediatr. Res. 19 (9), 879–886. 10.1203/00006450-198509000-00001 2413420

[B8] Korotko G. F. Baibekova G. D. (1989). Inhibition of pancreatic secretion by trypsin and trypsinogen. Fiziol. Zh. SSSR Im. I. M. Sechenova 74 (9), 1309–1315. 2463941

[B9] Laporte J. C. Tremolieres J. (1971). Hormonal regulation of the enzymatic secretion of the exocrine pancreas. Comptes rendus l'Académie Sci. 273, 1205–1207. 5001366

[B10] Liu H. X. Rajapaksha P. Wang Z. Kramer N. E. Marshall B. J. (2018). An update on the sense of taste in chickens: a better developed system than previously appreciated. J. Nutr. Food Sci. 8 (2), 686. 10.4172/21559600.1000686 29770259 PMC5951165

[B11] Lui J. C. May D. Miller D. Ouyang S. (1986). Cholecystokinin mediates feedback regulation of pancreatic enzyme secretion in rats. Am. J. Physiol. 250, 252–259. 10.1152/ajpgi.1986.250.2.G252 3953805

[B12] Moore C. X. Cooper G. J. S. (1991). Co-secretion of amylin and insulin from cultured islet beta-cells: modulation by nutrient secretagogues, islet hormones and hypoglycemic agents. Biochem. Biophys. Res. Commun. 179, 1–9. 10.1016/0006-291x(91)91325-7/ 1679326

[B13] Niknafs S. Roura E. (2018). Nutrient sensing, taste and feed intake in avian species. Nutr. Res. Rev. 31 (2), 256–266. 10.1017/S0954422418000100 29886857

[B14] Norton L. Shannon C. Gastaldelli A. Defronzo R. A. (2022). Insulin: the masterregulator of glucose metabolism. Metabolism 129, 155142. 10.1016/j.metabol.2022.155142 35066003

[B15] Pavlov I. P. (1951). Innervation of the pancreas. Ed. Leningr. USSR Acad. Sci. USSR 2 (1), 96–132.

[B16] Permyakov N. K. Podolsky A. E. Titova G. P. (1973). Ultrastructural analysis of the pancreas secretory cycle. Moscow: Medicine, 239.

[B17] Qaid M. M. Abdelrahman M. M. González-Redondo P. (2016). Role of insulin and other related hormones in energy metabolism—a review. Cogent Food and Agric. 2 (1). 10.1080/23311932.2016.1267691/

[B18] Rothman S. Leibow C. Isenman L. (2002). Conservation of digestive enzymes. Phsyological Rev. 82, 1–18. 10.1152/physrev.00022.2001 11773607

[B19] Saltiel A. R. Kahn C. R. (2001). Insulin signalling and the regulation of glucose and lipid metabolism. Nature 14 (6865), 799–806. 10.1038/414799a 11742412

[B20] Singer M. W. Niebergall-Roth E. (2009). Secretion from acinar cells of the exocrine pancreas: role of enteropancreatic reflexes and cholecystokinin. Cell Biol. Int. 33 (1), 1–9. 10.1016/j.cellbi.2008.09.008 18948215

[B21] Sliwowska J. H. Fergani C. Gawałek M. Skowronska B. Fichna P. Lehman M. N. (2014). Insulin: its role in the central control of reproduction. Physiol. Behav. 133, 197–206. 10.1016/j.physbeh.2014.05.021 24874777 PMC4084551

[B22] Vertiprakhov V. Grozina A. Fisinin V. Egorov I. (2018). The correlation between the activities of digestive enzymes in the pancreas and blood serum in chicken. Open J. Anim. Sci. 8, 215–222. 10.4236/ojas.2018.83016

[B23] Vertiprakhov V. G. (2022). Physiology of intestinal digestion in chickens (experimental approach). Moscow, Russia: K.A. Timiryazev Russian State Agrarian University - MSHA.

[B24] Vertiprakhov V. G. Borisenko K. V. (2019). Digestion and blood biochemical values of hens fed on the diets supplemented with exogenous protease. Sib. Her. Agric. Sci. 49 (2), 77–84. 10.26898/0370-8799-20192-10

[B25] Vertiprakhov V. G. Egorov I. A. (2016). The influence of feed intake and conditioned reflex on exocrine pancreatic function in broiler chicks. Open J. Anim. Sci. 6, 298–303. 10.4236/ojas.2016.64034

[B26] Vertiprakhov V. G. Grozina A. A. (2018). The estimation of pancreatic functionality in chicken using tryptic activity in blood serum. Veterinary 12, 51–54. 10.30896/0042-4846.2018.21.12.51-54

[B27] Vertiprakhov V. G. Grozina A. A. Dolgorukova A. M. (2016). The activity of pancreatic enzymes on different stages of metabolism in broiler chicks. Agric. Biol. 4, 509–515. 10.15389/agrobiology.2016.4.509eng

[B28] Vertiprakhov V. G. Grozina A. A. Fisinin V. I. Surai P. F. (2023). Adaptation of chicken pancreatic secretory functions to feed composition. World's Poult. Sci. J. 79 (1), 27–41. 10.1080/00439339.2023.2163042

[B29] Wang B. J. Cui Z. J. (2007). How does cholecystokinin stimulate exocrine pancreatic secretion? From birds, rodents, to humans. Am. J. Physiol. Regul. Integr. Comp. Physiol. 92 (2), R666–R678. 10.1152/ajpregu.00131.2006 17053097

[B30] Yoshida Y. Kawabata Y. Kawabata F. Nishimura S. Tabata S. (2015). Expressions of multiple umami taste receptors in oral and gastrointestinal tissues, and umami taste synergism in chickens. Biochem. Biophys. Res. Commun. 466 (3), 346–349. 10.1016/j.bbrc.2015.09.025 26361143

